# Molecular mechanisms of cooperative binding of transcription factors Runx1–CBFβ–Ets1 on the TCRα gene enhancer

**DOI:** 10.1371/journal.pone.0172654

**Published:** 2017-02-23

**Authors:** Kota Kasahara, Masaaki Shiina, Ikuo Fukuda, Kazuhiro Ogata, Haruki Nakamura

**Affiliations:** 1 College of Life Sciences, Ritsumeikan University, Kusatsu, Shiga, Japan; 2 Graduate School of Medicine, Yokohama City University, Kanazawa-ku, Yokohama, Kanagawa, Japan; 3 Institute for Protein Research, Osaka University, Suita, Osaka, Japan; Goethe-Universitat Frankfurt am Main, GERMANY

## Abstract

Ets1 is an essential transcription factor (TF) for several important physiological processes, including cell proliferation and differentiation. Its recognition of the enhancer region of the *TCRα* gene is enhanced by the cooperative binding of the Runx1–CBFβ heterodimer, with the cancelation of phosphorylation-dependent autoinhibition. The detailed mechanism of this interesting cooperativity between Ets1 and the Runx1–CBFβ heterodimer is still largely unclear. Here, we investigated the molecular mechanisms of this cooperativity, by using molecular dynamics simulations. Consequently, we detected high flexibility of the loop region between the HI2 and H1 helices of Ets1. Upon Runx1–CBFβ heterodimer binding, this loop transiently adopts various sub-stable conformations in its interactions with the DNA. In addition, a network analysis suggested an allosteric pathway in the molecular assembly and identified some key residues that coincide with previous experimental studies. Our simulations suggest that the cooperative binding of Ets1 and the Runx1–CBFβ heterodimer alters the DNA conformation and induces sub-stable conformations of the HI2–H1 loop of Ets1. This phenomenon increases the flexibility of the regulatory module, including the HI2 helix, and destabilizes the inhibitory form of this module. Thus, we hypothesize that this effect facilitates Ets1–DNA binding and prevents the phosphorylation-dependent DNA binding autoinhibition.

## Introduction

The regulation of gene expression by the specific binding of transcription factors (TFs) to regulatory *cis*-elements is an essential process for living organisms. Therefore, the elucidation of the molecular mechanisms of TF—DNA binding is a central issue in molecular biology. During the past decade, the advances of second-generation sequencing technologies have provided a bird’s eye view of the TF binding sites on the human genome, by using the ChIP-seq technique [1]. However, the atomic details of transcriptional regulation are still largely unknown. Although the three-dimensional (3D) structures of TFs—DNA complexes have shed light on their physical interactions, the regulation of gene expression is too complicated to be understood from the static views of the molecular structures. TFs—DNA interactions are modulated via a variety of mechanisms, such as post-translational modifications, folding—unfolding transitions of disordered regions in the TFs, and multimeric interactions with other partner TFs. Therefore, the elucidation of the regulation mechanisms of TFs—DNA interactions is a long-standing conundrum in this field.

An illustrative example is the v-ets erythroblastosis virus E26 oncogene homolog 1 (Ets1), which plays important roles in several essential pathological phenomena, such as cancer development and autoimmunities [2,3]. Ets1 is composed of six domains: the N-terminal, pointed, transactivation, D, ETS, and F domains [4]. The main domain involved in the DNA recognition is the well-conserved ETS domain, consisting of seven helices referred to as HI1, HI2, and H1 through H5, and the four-stranded β-sheet. The H3 helix, also known as the recognition helix, which is part of a winged helix-turn-helix motif, is buried within the major groove of the DNA and recognizes the signature sequence, 5’-GGA(A/T)-3’. There are three major mechanisms for the regulation of DNA binding. i) The formation of the tightly packed module by the four short helices, HI1, HI2, H4, and H5, called the “inhibitory module”, inhibits DNA binding [5–7]. ii) The function of Ets1 can also be inhibited by phosphorylation within the disordered serine-rich region (SRR), on the N-terminal side from the ETS domain. The phosphorylation of serine residues in this region; *i*.*e*., Ser251, Ser282, and Ser285, additively down-regulates the DNA binding [8]. iii) Furthermore, Ets1 recognizes the regulatory elements as a result of cooperative binding interactions with other partner TFs [9]. For example, Ets1 recognizes the promoter region of the *mb-1* gene by cooperative binding with paired box protein-5 (Pax5), also known as B cell-specific activator protein. Although the sequence of this promoter region (5’-GGAG-3’) slightly differs from the standard consensus sequence of Ets1 (5’-GGA(A/T)-3), the cooperative binding with Pax5 allows Ets1 to recognize this region, due to a rotamer change of the Ets1 Tyr395 residue [7,10]. Another example is the promoter region of the *stromelysin-1* gene, which is recognized by homo-dimerized Ets1 in the head-to-head configuration [11–13].

In another case, the binding of Ets1 with the enhancer region of the *T-cell antigen receptor α/β* (*TCRα/β*) gene is supported by cooperative binding with the Runt-related transcription factor-1 (Runx1)–the core binding factor β (CBFβ) heterodimer. Shiina *et al*. recently reported the crystal structures of the Runx1–CBFβ–Ets1–DNA quaternary complexes [14,15]. Interestingly, in this complex, even when the SRR is phosphorylated, the Ets1–DNA binding is not inhibited. The cancelation of the phosphorylation-dependent inhibition was observed in this complex form but not observed in any other known complexes [14]. The 3D structures of this complex clearly revealed the absence of direct contacts between Ets1 and the partner TFs, and thus some type of allosteric effect plays a key role for this robust resistance to the phosphorylation-dependent inactivation. In terms of the 3D structure, a unique feature of this quaternary complex is the fact that the HI2 helix is partly unfolded, which means that the HI2 helix in this assembly is shorter than those in other forms of Ets1. This implies that the conformational change in HI2 is important for the cooperativity. As this region, previously called the inhibitory module, also conducts the activation of DNA binding, we refer to it as the “regulatory module (RM)” hereafter, according to the report by Shiina *et al*. [14]. In order to understand its molecular mechanism, the dynamics of this molecular assembly should be investigated at the atomic level.

Toward this goal, the molecular dynamics (MD) method is a promising technique. Over the past decade, this method has been applied to study the mechanisms of Ets1–DNA binding. Reddy *et al*. investigated the conformational dynamics of Ets1 with two different DNA sequences [16]. Kamberaje and Vaart studied the correlative motions between the H1 helix and DNA, which play important roles for the DNA binding regulation elicited by the RM [17]. Karolak and Vaart presented the results of the replica exchange MD simulations for some helices composing the RM, and suggested that specific hydrophobic contacts stabilize the RM [18]. Recently, we reported the development of the new data analysis method, named the “multi-modal dynamic cross correlation (mDCC)” method, which is a variant of the conventional dynamic cross correlation (DCC) method, and discussed the results of its application to the homo-dimerized Ets1–DNA complex [19]. While the multi-modal behavior of atomic motions, such as rotamer changes of amino acid side-chains tends to be overlooked in conventional methods, our mDCC approach can suitably characterize multi-modal behavior by taking advantage of a Bayesian statistics-based pattern recognition technique. We thus found some transient interactions appearing at the intermolecular interfaces of Ets1–Ets1 and Ets1–DNA [19].

Here, we describe the molecular mechanism of the cooperative binding in the Runx1–CBFβ–Ets1–DNA quaternary complex, determined using MD simulations. We performed a total of 1.4 μs simulations for the five molecular models, derived from the crystal structures reported by Shiina *et al*. [14]; *i*.*e*., the quaternary complex, an Ets1–DNA complex, an isolated DNA, and two kinds of quaternary complexes with mutations, K167A in Runx1 and Y329A in Ets1. These mutants were previously shown to reduce the cooperativity between Ets1 and the Runx1–CBFβ heterodimer [14]. Comparisons of the dynamic features among these molecular models provided insight into the mechanism of cooperative binding with the Runx1–CBFβ heterodimer. For analyses of the simulation trajectories, we took advantage of a pattern recognition technique and complex network analysis, by using the mDCC method [19,20]. Our analyses revealed the unique dynamics of the partly unfolded HI2 helix and the adjacent HI2–H1 loop in the quaternary complex.

## Results

### Dynamic behavior of the regulatory module of Ets1

**Fig 1:**
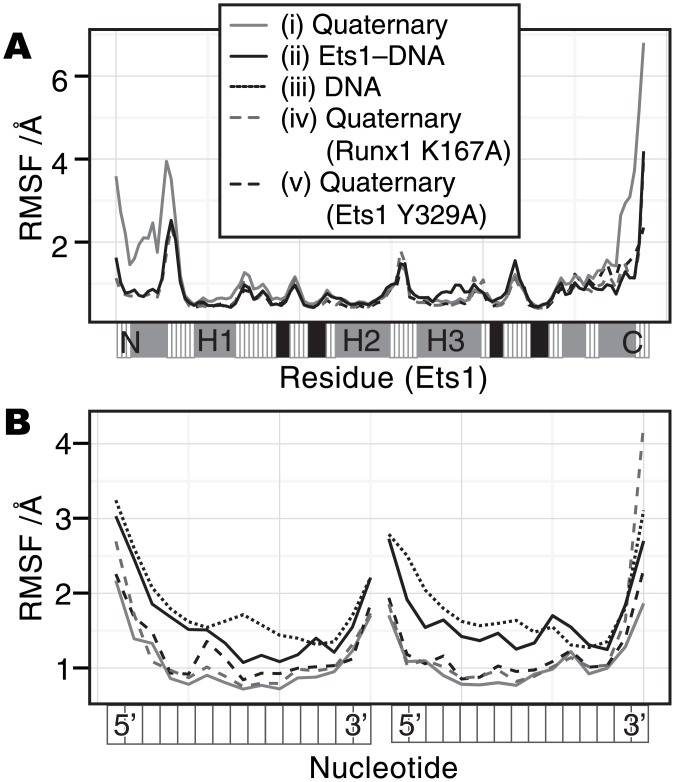
The RMSF value of each residue. (A) The RMSF values of the Cα atoms in Ets1. (B) The RMSF values of the phosphorus atoms in DNA. The horizontal axes represent each residue of the molecule from the N-terminus to the C-terminus for Ets1, and from 5’ to 3’ for the DNA. The grey solid line, black solid line, grey dashed line, black dashed line, and black dotted line indicate the results from (i) the quaternary complex, (ii) the Ets1–DNA complex, (iii) the isolated DNA, (iv) the K176A model, and (v) the Y329A model, respectively. The bar below the plot in panel (A) is a secondary structure guide of Ets1: grey, black and white indicate α-helix, β-strand, and others, respectively. For the RMSF calculations of each molecule, the trajectories were superimposed only on the backbone atoms of the molecule, and the other molecules in the model were ignored.

We simulated the dynamical behavior of five molecular models: (i) the (wild-type) quaternary model, (ii) the Ets1–DNA model, (iii) the DNA model, (iv) the quaternary (Runx1 K167A) model, and (v) the quaternary (Ets1 Y329A) model. The time course of the root mean square deviation (RMSD) of each molecule in each model (S1 Fig) demonstrated that there are no significant conformational changes in the wild-type quaternary complex except for Ets1, which sometimes exhibits an RMSD of nearly 4 Å (panel A, red plot). This large deviation in the quaternary complex can be explained, by focusing on the root mean square fluctuation (RMSF) of each residue. Fig 1A shows that the two regions, from the N-terminus to the H1 helix and the C-terminus of Ets1 largely fluctuate in the quaternary complex, as compared to the other models (the RMSFs of the other molecules are shown in S2 Fig). In particular, the conformation of the HI2–H1 loop deviates from that in the crystal structure, and the hydrogen bond between the Gly333 amide group and the C112 phosphate is repeatedly formed and disrupted (S3 Fig). This interaction has been identified as one of the key features of the cooperative binding in this complex, based on the fact that the single mutants G333P and P334G reduce the binding affinity of phosphorylated Ets1 to the Runx1–CBFβ heterodimer [14]. The simulation results suggest that this region largely fluctuates and the Gly333–C112 interaction is transiently deformed.

**Fig 2:**
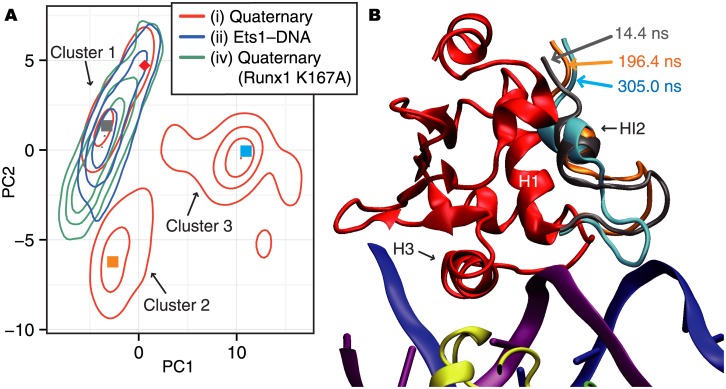
The PCA analysis of the N-terminal region of Ets1. (A) A 2D contour map of the conformational space. The red, green, and blue lines indicate the results of the wild-type quaternary complex, the quaternary Runx1 K176A model, and the Ets1–DNA complex, respectively. The density distribution of conformations sampled from each trajectory is projected onto the 2D space defined by the first and second principal components (PC1 and PC2). From this map, the three clusters, clusters 1, 2, and 3, were defined. Arbitrarily chosen representative snapshots of each cluster, taken at 14.4 ns, 196.4 ns, and 305.0 ns for clusters 1, 2, and 3, respectively, are plotted as filled squares colored cyan, pink, and green, respectively. The conformation in the crystal structure (PDB ID: 3wts) is plotted as the red diamond. (B) A 3D image of the quaternary complex at the three representative snapshots. Only the N-terminal region, which is considered in the PCA analysis, is shown for the three snapshots, with the same color scheme as in panel (A). The structures of the other regions were taken at 305.0 ns at cluster 3.

In order to analyze the conformational changes of the fluctuating N-terminal region of Ets1 in the quaternary complex, the principal component analysis (PCA) was performed on the Cartesian coordinates of the Cα atoms of the region, from the N-terminus to the HI2–H1 loop (Val320 through Gln336). Fig 2A shows the density distribution in the conformational space projected on the first and second principal component axes (PC1 and PC2), which have contribution rates of 40.3% and 20.2%, respectively. The conformations of the N-terminal region of Ets1 in the wild-type quaternary complex fall into three clusters, named clusters 1, 2, and 3 (Fig 2A). Examples of the structures in each cluster are shown in Fig 2B, and their positions on the PC1–PC2 plane are plotted as filled squares in Fig 2A. In the Ets1–DNA and quaternary (Runx1 K167A) models, only one cluster overlaps with cluster 1 of the quaternary complex. In other words, only the wild-type quaternary complex deviates from the conformations in the crystal structure during the simulation. The time course of the PC1 and PC2 values in the wild-type quaternary complex revealed that the conformation mainly belongs to cluster 1 in the first 90 ns, then transits to cluster 2 for the next 110 ns. Subsequently, the conformations in cluster 3 appear and persist until 320 ns. Then, the system repeats the transitions between clusters 2 and 3 (S4 Fig and S1 Movie). The HI2–H1 loop in cluster 2 (orange ribbon in Fig 2B) is slightly displaced upward as compared with cluster 1 (grey ribbon). In contrast, cluster 3 (cyan ribbon) has a very different conformation from those of the other two clusters. The HI2–H1 loop in clusters 1 and 2 curls upward; however, that in cluster 3 is directed downward and Ser332 interacts with C112 of the DNA. (See also S3 Fig for a comparison between the crystal structure and the structure in cluster 3.)

This Ser332–C112 interaction is a remarkable feature of cluster 3. The PC1 value is negatively correlated with the interatomic distance between the backbone carboxyl carbon atom of Ser332 (referred to as “Ser332:C”, hereafter) and the C5’ atom of C112 (the Pearson correlation coefficient is -0.71 in the quaternary complex; the time courses are shown in S5A Fig). Since the HI2–H1 loop largely fluctuates and the Ser332–C112 interaction is transient, the conventional DCC detects only a weak correlation between them (DCC value is 0.178). In contrast, mDCC showed a highly positive correlation (mDCC value is 0.501). For this interacting pair, since the DNA is rigid as compared to the flexible HI2–H1 loop, C112:C5’ has a uni-modal distribution and Ser332:C falls into five modes, with probabilities of 0.456, 0.338, 0.110, 0.0741, and 0.0621. Among them, the second and fourth modes interact with C112. In other words, the Ser332–C112 interaction is formed during approximately 41% of the trajectory. This transient interaction is a CH–π interaction with the peptide backbone π plane. In addition, the side-chain hydroxyl group transiently interacts with the backbone phosphate of T113 (see S5B Fig for the time course of the interatomic distances of Ser332:Oγ–T113:OP1). This interaction also supports the conformational changes of the loop region. Although we experimentally assessed the effects of S332 by introducing the S332A mutation and measuring the Ets1–DNA binding affinities with and without the Runx1–CBFβ heterodimer, the impact of this mutation is not significant (See S1 Text and S6 Fig for experimental details). As the backbone atoms of Ser332 also interact with C112 of the DNA chain during the simulation, the mutation might not eliminate the cooperativity.

Conformational fluctuations in the RM with the deformation of several hydrophobic contacts are uniquely observed in the quaternary complex model (S2 Movie). For example, the average (and standard deviation) of the distances between Ile321–Leu429 is 9.04 Å (3.08 Å), in the quaternary complex, while it is 4.39 Å (0.672 Å) in the Ets1–DNA complex. The time courses of the interatomic distances of Ile321–Leu429 and Ile321–Tyr424 are shown in S7 Fig, and the structure of the RM is shown in S2 Fig. These results suggest that the cooperative binding with the Runx1–CBFβ heterodimer induces large fluctuations of the HI2–H1 loop and the RM, which form many transient interactions.

### Relationship between DNA conformation and TF binding

As mentioned above, the binding with the partner TFs, the Runx1–CBFβ heterodimer, affects the motion of Ets1, in spite of the fact that there are no direct interactions. This implies that the DNA molecule mediates the effects of the Runx1–CBFβ heterodimer on Ets1. The RMSF values of the backbone phosphorus atoms of DNA (Fig 1B) revealed that the DNA rigidity increases in the following order: the isolated DNA (the average RMSF of the phosphorus atoms is 1.98 Å), the Ets1–DNA complex (1.78 Å), the mutated quaternary complexes (1.31 Å for Runx1(K167A) and 1.26 Å for Ets1(Y329A)), and the wild-type quaternary complex (1.14 Å).

**Fig 3:**
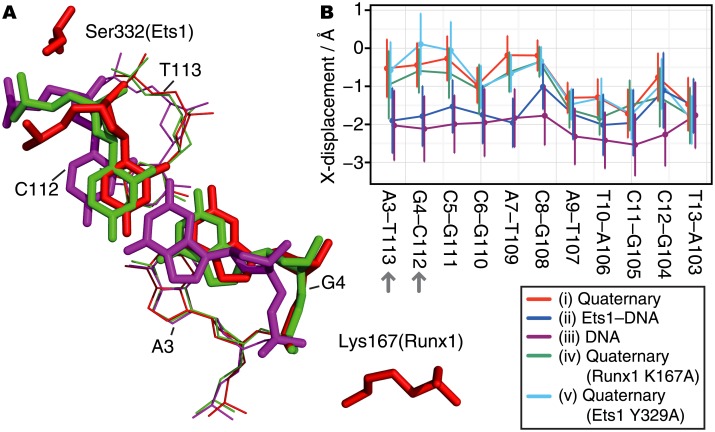
Changes in DNA conformations around the G4–C112 base-pair. (A) 3D structures of the G4–C112 base-pair taken from the three simulations: the wild-type quaternary complex (red), the quaternary complex with the Runx1 K167A mutant (green), and the isolated DNA (purple). The snapshots were taken at the times when the X-displacement parameter was near the average value for each model. The three structures are superimposed, based on the adjacent base-pair (A3–T113; the thin lines). (B) The averages and standard deviations of the X-displacement parameter in the wild-type quaternary complex (red), the Ets1–DNA complex (blue), the isolated DNA (purple), the quaternary complex with Runx1 K167A mutant (green), and the quaternary complex with Ets1 Y329A mutant (cyan).

The details of the conformational differences in the DNA were assessed with the Curves+ software [21]. The most remarkable difference among the simulation models is found in the X-displacement parameter, which indicates the displacement of a base-pair toward the major and minor grooves, corresponding to positive and negative values, respectively. As shown in Fig 3, the binding of the Runx1–CBFβ heterodimer and Ets1 to DNA affects the DNA conformations. First, the isolated DNA shows the lowest X-displacement values. Second, in the Ets1–DNA complex, while the X-displacements in the first half of the base-pairs in Fig 3B (5’-AGCCAC-3’) are comparable to those of the isolated DNA, the X-displacements in the remaining half (5’-ATCCT-3’) are significantly larger and comparable to those of the quaternary complex. The first and last halves of the DNA correspond to the binding sites for Runx1 and Ets1, respectively. This implies that the binding of these TFs shifts the X-displacement geometry toward the major groove. Among the three quaternary complexes, containing either the wild-type, Runx1(K167A), or Ets1(Y329A) mutant components, the lack of the N-terminal part of Ets1 in the Y329A model and the loss of the direct interaction between G4 and Lys167(Runx1) in the K167A model cause subtle conformational changes in some base-pairs in the first half (5’-AGCCAC-3’).

### Correlative motions in the Runx1–CBFβ–Ets1–DNA complex

**Fig 4:**
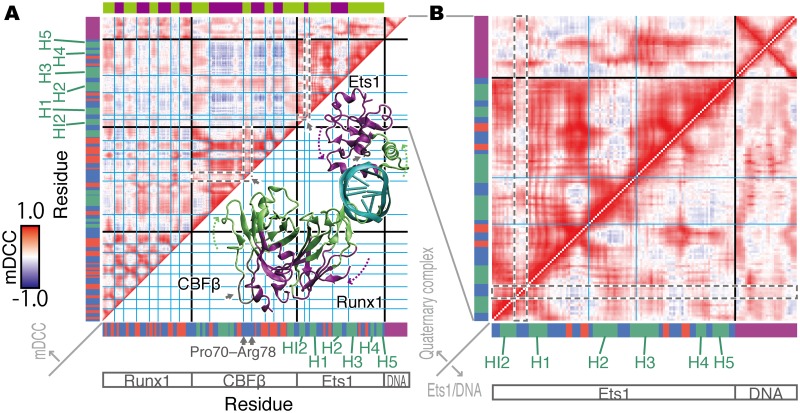
The mDCC correlation maps. (A) The correlation map of the quaternary complex. The horizontal and vertical axes indicate each residue from the N- to C-termini of Runx1, CBFβ, and Ets1, and the 5’- to 3’-termini of the DNA chains. The colored bars along the axes indicate the secondary structures of each residue: green, red, and blue indicate α-helix, β-strand, and others, respectively; and nucleotides are colored purple. In the heatmap, the graduation from blue to red corresponds to mDCC values from -1.0 to 1.0. The grid drawn with cyan lines highlights the checkered pattern on the map. It divides the residues into the two groups colored green and purple in the color bar at the top of the map, and in the 3D structure of the molecular complex overlaid on the lower triangle of the map. The grey arrows indicate the flexible loops discussed in the main text. The region corresponding to these loops on the heatmap are marked as grey dashed lines. (B) The correlation maps focusing on Ets1 and DNA in the quaternary complex (the upper-triangle) and in the Ets1–DNA complex (the lower-triangle). The upper-triangle of panel B is identical to part of the map in panel (A). The grey dashed lines highlight the HI2–H1 loop, discussed in the main text.

In order to elucidate the effects of the partner TFs on the Ets1 dynamics, we next analyzed the correlations of atomic motions for all-to-all pairs of atoms, by using the mDCC analysis method [19,20]. The correlation map, which depicts the correlation coefficients, or mDCC values, for every pair of residues, calculated from the trajectory of the wild-type quaternary complex, is shown in Fig 4A. Residue pairs colored red, white, and blue have positive correlation, weak (or non-) correlation, and negative correlation, respectively. The correlation map shows a checkered pattern, indicating that the residues can be divided into two groups (the green and purple regions on the colored bar at the top of the figure; the borders of the groups are highlighted by cyan lines on the map). Residue pairs within the same group tend to move toward the same direction. In contrast, two residues from different groups tend to move in opposite directions. These two groups, green and purple, correspond to the near- and far-sides of the molecules from the DNA, as shown in the 3D model overlaid on the lower triangle of the correlation map (Fig 4A). This correlation pattern reflects the flipping motion of TFs around the helix axis of the DNA (S3 Movie). The HI2–H1 loop of Ets1 and the loop consisting of Pro70 through Arg78 of CBFβ (the areas surrounded by grey dashed lines in the correlation map, Fig 4A) have more whitish colors. In other words, they independently fluctuate from the flanking helices. These observations suggest that binding with the partner TFs, Runx1 and CBFβ, induces the flipping global motions of Ets1, except for the flexible loops.

The mDCC maps between the quaternary complex (the upper triangle) and the Ets1–DNA complex (the lower triangle) are compared in Fig 4B. They illustrate that many intramolecular positive correlations of Ets1 in the quaternary complex become weak or non-correlated by removing the partner TFs, which may be due to the loss of the flipping motions. Most remarkably, the motions of the HI2–H1 loop and its flanking regions are altered. The HI2–H1 loop behaves independently from the flanking regions in the quaternary complex. In contrast, the motions of the loop are similar to those of the flanking regions in the Ets1–DNA complex. This result implies that the binding of the partner TFs with DNA facilitates the separation of the motions of the HI2–H1 loop from those of the flanking helices, and this may be due to the transient interactions between this loop of Ets1 and the DNA. In addition, the binding of the partner TFs with DNA also facilitates the correlative motions between the Ets1 HI2–H1 loop and the DNA, especially the first five base-pairs (from G1–C115 to C5–G111).

### Network analysis of the correlative motions in the Runx1–CBFβ–Ets1–DNA complex

We visualized the connections of the atomic interactions as a network diagram, in order to analyze the mechanism of allosteric regulation by the partner TFs. Since allosteric effects are generally considered to be propagated through atomic contacts [22], we drew the networks by picking the residue pairs with highly positive correlations (mDCC ≥0.5) and atomic contacts (distance between centers of modes <5.0 Å) from the mDCC maps. In the networks, we assessed the importance of each residue, by using the betweenness measure (the top 15 highest betweenness nodes in each molecule are shown in S2 Table). Importantly, the interaction networks include information about the multi-modal dynamic features, by taking advantage of the mDCC method. Even when the interactions between residues only occurred in part of the trajectory, the mDCC method detects such transient interactions and draws edges between them.

As a result, the betweenness measurement successfully detected known important residues. Some of the H3 recognition helix residues, Arg391, Tyr395, Leu393, Tyr396, Tyr397, and Tyr386, are ranked as the first, third, fourth, fifth, eleventh, and twelfth highest betweenness residues in Ets1. While Leu337, which is an essential residue for the autoinhibition [23], is not ranked in the top 15 residues, its neighbors, Gln336 and Trp338, are at the tenth and second positions, respectively. Similarly, while Gly333 and Pro334 have rather low betweenness values, the adjacent Ser332 has the sixth highest value. In addition, Trp375, within the hydrophobic core of Ets1, is ranked as the eighth. For Runx1, some key residues for the DNA binding were previously identified; *i*.*e*., single point mutations of the following residues reduce DNA binding: Arg80, Arg83, Arg135, Arg139, Arg142, Lys167, Thr169, Asp171, Arg174, and Arg177 [24]. In our analysis, the first four residues have high betweenness values (fifth, seventh, first, and twelfth highest values in Runx1, respectively). As our model uses Runx1 truncated at position 177 and capped with an N-methyl group, the interactions of Arg177 cannot be investigated. It should be mentioned that the truncation of the polypeptide at position 176 might lead to the relatively low betweenness values for these important residues. In addition, the network with the other criteria (edges with mDCC ≥0.7) also successfully highlighted the important residues (S2 Text and S3 Table).

**Fig 5:**
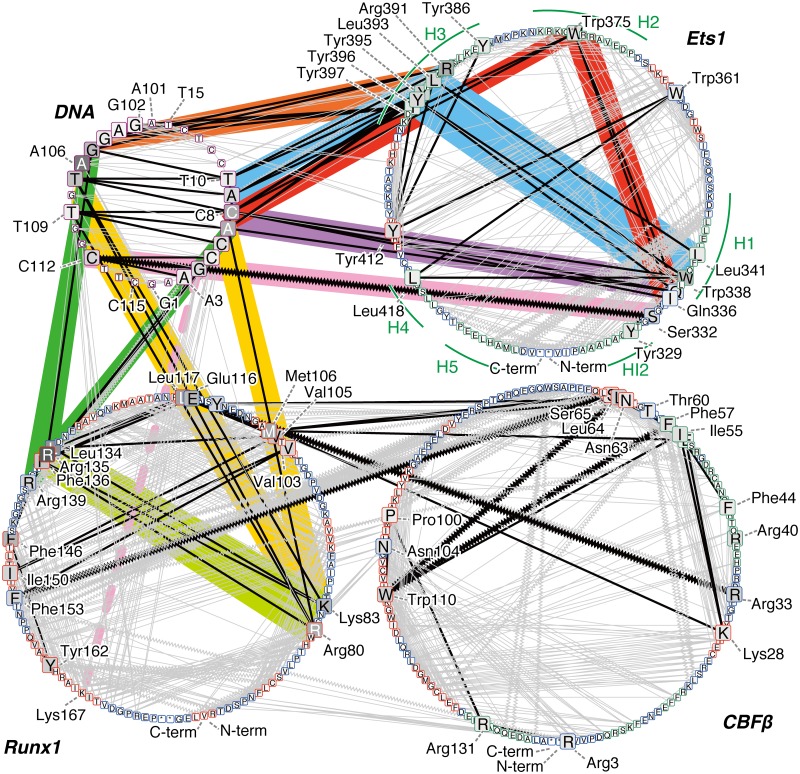
The correlation network of the wild-type quaternary complex. Each node indicates each residue, labeled with the one-letter codes for amino acids and nucleotides. An asterisk “*” denotes the termini of polypeptides, *i*.*e*., N-methyl and acetyl groups. The circles correspond to DNA (upper-left circle), Ets1 (upper-right), Runx1 (lower-left), and CBFβ (lower-right). Nodes are aligned in the sequence order for each molecule in the counter-clockwise direction, starting from the bottom of each circle. The colors of the nodes indicate the betweenness values: higher values are darker. In particular, the top 15 highest betweenness residues in each molecule are shown as large squares. The colors of the node borders indicate the secondary structures of the residues: green, red, and blue represent α-helix, β-strand, and others, respectively; nodes in DNA are shown as a purple border. Edges are drawn between nodes with highly positive correlations (mDCC ≥0.5) with contact (distance between the centers <5Å). Edges between the top 15 betweenness residues are colored black, and other edges are grey. Zigzag edges indicate transient interactions, meaning that DCC <0.5 but mDCC ≥0.5. Some edges that are particularly discussed in the main text are highlighted with a colored background.

**Fig 6:**
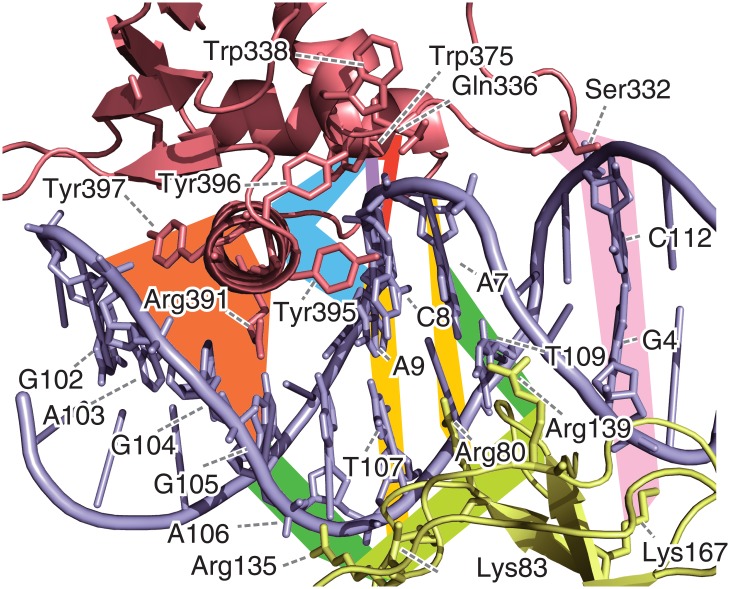
The 3D structure of the wild-type quaternary complex, focusing on the Ets1–DNA—Runx1 interface at 305.0 ns. The residues with the top 15 highest betweenness values in each molecule are shown as sticks. The colored background bands correspond to the lines in Fig 5.

In the interaction network, the connections of these high betweenness residues can be considered as a scaffold for the network (the black edges in Fig 5). The H3 helix, which is buried within the major groove of the DNA, interacts with some bases, including A106, C8, A7, G105, and A9 (highlighted in orange and cyan in Fig 5). The nucleotides C8 and A9 interact with Trp375 in the H2 helix (red) and the N-terminus of the H1 helix, which is composed of Trp338, Gln336, and Leu341 (purple). Furthermore, the N-terminus of H1 interacts with H2 (red) and H3 (cyan). In summary, the relationships of the Ets1–DNA interactions can be represented by two triangles sharing one edge, which are the purple—red—red and purple—cyan—cyan triangles. They share the purple edges, which means that the interactions between C8 and the H1 helix (Gln336~Trp338) are important to establish the connections of the network. This sub-network (highlighted in cyan, red, purple, and orange in Figs 5 and 6) is conserved in the Ets1–DNA complex (S8 Fig) and the Ets1 homo-dimer—DNA complex on the stromelysin-1 promoter region, which was analyzed in our previous study [19]. The vertices of these triangles on the DNA, G4–T10 and G102–T107, are the interfaces between Ets1 and Runx1. In Runx1, Arg135 and Arg139 interact with G105–A107 and G4–C6, respectively (green), while Arg80 interacts with A106–T107, and Lys83 interacts with T107, T109, and A7 (yellow). These two interface regions of Runx1 mutually interact (lime green). In addition to these interfaces, Ser332 of Ets1 transiently interacts with C112 upon the large fluctuation of the HI2–H1 loop. Ser332 has the sixth highest betweenness in Ets1 (pink). The base-pair partner of C112, G4, interacts with Lys167 of Runx1 (the dashed line), which was identified as an important residue for cooperative binding with Ets1 by the K167A mutation experiment [14], although it does not exhibit high betweenness.

This network suggests the existence of an allosteric pathway, in which the effects of the Runx1–CBFβ heterodimer propagate to Ets1 via the DNA. While the majority of the Ets1–DNA interactions do not differ much between the quaternary and Ets1–DNA complexes (edges highlighted in red, cyan, purple, and orange in Fig 5 and S9 Fig), the interface bases also interact with Runx1 in the quaternary complex (yellow and green lines). These interactions seem to stabilize the Ets1–DNA interactions. In addition, the Ets1–DNA interaction at Ser332–C112, which is stabilized by Lys167 of Runx1, uniquely appears in the quaternary complex (pink lines).

## Discussion

In this study, we simulated several molecular models originating from the crystal structure recently solved by Shiina *et al*. [14] in order to elucidate the molecular mechanisms underlying the cooperative binding of Runx1, CBFβ, and Ets1 to the *TRCα* enhancer region. According to the comparison between the motions of the quaternary complex and that of the Ets1–DNA complex, the binding of the Ets1–DNA complex with the partner TFs induces flipping domain motions in Ets1 around the DNA helix axis (Fig 4 and S3 Movie). In addition to the impact of the cooperative binding on the entire motions of the molecular assembly, we found that the N-terminal HI2 helix and the HI2–H1 loop region of Ets1 largely fluctuate when the Runx1–CBFβ heterodimer binds to the enhancer (Fig 1). The large RMSF values reflect the subtle shift of the loop from the ETS domain (cluster 2 in Fig 2) and the conformational change of the loop region induced by the transient interaction between Ser332 and C112 (cluster 3 in Fig 2 and S1 Movie). As a result, the orientation of the HI2 helix of Ets1 is altered and the hydrophobic packing at the RM is transiently disrupted (S6 Fig and S2 Movie). As demonstrated by previous mutation experiments, the tightly packed structure of the RM is a key feature for the autoinhibition in Ets1, and mutations of the bulky hydrophobic side-chains facilitate the DNA binding [6,7]. In the simulation for the wildtype quaternary complex, the RM becomes destabilized. This instability of the RM is not observed in the simulation of either the Ets1–DNA model or the K167A mutant-containing quaternary complex. Thus, we conclude that the cooperative binding of Ets1 and the Runx1–CBFβ heterodimer induces the fluctuation of the RM, and facilitates the Ets1–DNA binding. The reduction of the cooperativity in Runx1 K167A was experimentally observed by Shiina *et al*. [14].

Here, we propose the allosteric mechanism of the cooperativity of TFs on the enhancer region. Note that while our network analysis shows correlations between residues, it does not suggest causality (the edges in the network are undirected). However, knowledge from the previous assays and the dynamics observed in the simulation trajectories provides insight into the molecular mechanisms. The network analysis illustrates that Ets1 communicates with the Runx1–CBFβ heterodimer through the DNA (Figs 5 and 6). The communication between Lys167 of Runx1 and Ser332 of Ets1 is mediated by the G4–C112 base-pair. The conformation of this base-pair is significantly altered upon binding with the partner TFs (Fig 3). In addition, the base A106 is one of the keystones of the cooperativity, because this base exhibited a very high betweenness value due to its direct interactions with both the H3 recognition helix of Ets1, and Lys83 and Arg135 of Runx1 (Fig 6).

In summary, the binding of the Runx1–CBFβ heterodimer stabilizes specific DNA conformations. These conformational changes facilitate the transient interactions between Ser332 of Ets1 and C112, which stabilize the sub-stable alternative conformations of the HI2–H1 loop. Consequently, the orientation of HI2 is altered, and it partly disrupts the hydrophobic packing in the RM. This phenomenon facilitates Ets1–DNA binding, by impairing the inhibitory effects of the RM.

Since the facilitation of Ets1–DNA binding counteracts the inhibitory effect of SRR phosphorylation, we hypothesize that the flexible motion of the RM, induced by the cooperative binding with the Runx1–CBFβ heterodimer, plays a key role for the robustness of this assembly against the phosphorylation-dependent inhibition. While we have no direct information about the dynamics of the disordered region, due to the lack of the unfolded HI1 helix and the SRR in our simulation models (Ets1 in the models consists of amino acids 319–436), we propose the following scenario. The phosphorylated SRR could inhibit the Ets1–DNA binding by adopting somewhat inhibitory structures; for instance, the DNA binding of the recognition helix can be competitively inhibited by the formation of direct contacts between the phosphor-serine and the recognition helix. Previously, the interactions between the phosphorylated SRR and the recognition helix were analyzed by Desjardins *et al*., based on NMR experiments [25]. Their analysis suggested that the destabilization of this inhibitory state reduces the phosphorylated SRR-dependent inhibition. Since the disordered SRR is directly connected to the N-terminal side of the RM, an increase in the flexibility of the RM can augment the conformational diversity of the SRR, thus reducing of the relative population of the inhibitory state in the conformational ensemble. In order to assess this hypothetical scenario, simulations with models including the SRR will be useful.

## Materials and methods

### Simulation models and simulation conditions

Five molecular models, based on the crystal structure solved by Shiina *et al*. [14], were simulated in this study: (i) the Runx1–CBFβ–Ets1–DNA quaternary complex (PDB ID: 3wts), (ii) the Ets1–DNA complex prepared by removing the Runx1–CBFβ heterodimer from the quaternary complex, (iii) the double-stranded DNA isolated from the quaternary complex, (iv) the Runx1(K167A)–CBFβ–Ets1–DNA complex (PDB ID: 3wtw), and (v) the Runx1–CBFβ–Ets1(Y329A)–DNA complex (PDB ID: 3wtx). For simplicity, we call them (i) the (wild-type) quaternary model, (ii) the Ets1–DNA model, (iii) the DNA model, (iv) the quaternary (Runx1 K167A) model, and (v) the quaternary (Ets1 Y329A) model, respectively, hereafter. The missing region in CBFβ, Ser72 through Thr80 (SWQGEQRQT), was built by using the Spanner web-server [26]. The sequences of the double-stranded DNA in these models are 5’-GAAGCCACATCCTCT-3’ and 5’-AGAGGATGTGGCTTC-3’ and correspond to chains D and E in the PDB model (PDB ID: 3wts). The positions of the bases are denoted as 1 to 15 for the first chain and 101 to 115 for the second one. In all models, the molecules were bathed into a 150 mM NaCl solution. Details of these simulation models are summarized in S1 Table.

For MD simulations, the energy optimization and equilibration runs were performed, using the Gromacs software [27]. The successive production runs were performed, using the myPresto/psygene-G software [28]. Amber parm99SB [29] with bsc0 parameters [30] was applied for the force-field of proteins and DNA. For the solution, the ion parameters determined by Joung and Cheatham [31] and the TIP3P water model [32] were used. For fast calculations of the electrostatic interactions, we used the zero-dipole summation (ZD) non-Ewald method [33–35], with the damping parameter α = 0. The accuracies of the ZD method and its generalization, *i*.*e*., the zero-multipole summation method [36], have been extensively evaluated over the past several years, not only for periodic systems, such as bulk water and liquid ions [37], but also for heterogeneous systems, such as soluble proteins, membrane proteins, and DNA [38]. The production runs of MD simulations were performed under the constant temperature (300 K) with a 1.0 fs integration time step, and the SHAKE algorithm [39] was applied to constrain the length of the covalent bond for an individual hydrogen atom.

### Analysis methods

The simulation trajectories were analyzed with the mDCC method. As the details of the method were thoroughly described in our previous papers [19,20], here we provide a brief description. In order to elucidate the molecular mechanisms of the cooperativity between TFs without direct contacts, we focused on the correlations of atomic motions. The dynamic cross correlation (DCC) method has been extensively applied to quantify the correlations of motions between pairs of atoms [40]. The DCC value between the *i*-th and *j*-th atoms is formalized as:

$$DCC\left(i,j\right)=\frac{{\langle \Delta r_{i}(t)\cdot \Delta r_{j}(t)\rangle }_{t}}{\sqrt{{\langle {\left\|\Delta r_{i}(t)\right\|}^{2}\rangle }_{t}}{\sqrt{\langle {\left\|\Delta r_{j}(t)\right\|}^{2}\rangle }}_{t}}$$ (1)

$$\Delta r_{i}(t)=r_{i}(t)-{\langle r_{i}(t)\rangle }_{t},$$ (2)

where **r**_i_(t) denotes the Cartesian coordinate of the *i*-th atom at time *t*, and the bracket 〈⋅〉_*t*_ indicates the ensemble average. In this method, the motions of atoms are characterized as the deviations from the averaged coordinate. However, atoms can show multi-modal behavior. For example, the flipping of an amino acid side-chain can generate a bi-modal distribution of atomic coordinates, corresponding to two stable rotamers. The average of such a multi-modal distribution is sometimes meaningless for the characterization of atomic motions.

In contrast, the mDCC method explicitly considers the multi-modal behavior of atoms by applying a pattern recognition technique [41] to classify the spatial distribution of coordinates. The algorithm automatically defines “modes” of atomic motions from the ensemble of the Cartesian coordinates. Utilizing these modes, the mDCC method measures the correlations between the *k*-th mode of the *i*-th atom and the *l*-th mode of the *j*-th atom with the following equations:

$$mDCC(i,j;k,l)=\frac{{\langle w_{i,j;k,l}(t)\left(\Delta r_{i,k}(t)\cdot \Delta r_{j,l}(t)\right)\rangle }_{t}}{\sqrt{{\langle w_{i,j;k,l}(t){\left\|\Delta r_{i,k}(t)\right\|}^{2}\rangle }_{t}}\sqrt{{\langle w_{i,j;k,l}(t){\left\|\Delta r_{j,l}(t)\right\|}^{2}\rangle }_{t}}}$$ (3)

$$w_{i,j;k,l}(t)=p_{k}(r_{i}(t))p_{l}(r_{j}(t)),$$ (4)

$$\Delta r_{i,k}(t)=r_{i}(t)-\mu_{k}.$$ (5)

The mDCC differs from the conventional DCC in the following two points: first, motions of atoms are characterized as deviations from the center of mode ***μ***_*k*_, and second, the deviations are weighted by *w*_*i*,*j;k*,*l*_(t), which is the joint probability for the events in which the *i*-th and *j*-th atoms belong to the *k*-th and *l*-th modes, respectively. Since the modes are defined as a probabilistic model, described as the Gaussian mixture distributions, the probability of the assignment of the *i*-th atom into the *k*-th mode at time *t*, *p*_*k*_(**r**_i_(t)), is formally defined. Note that the mDCC value is limited in the range from -1.0 to 1.0.

With the mDCC method, we analyzed all modes of all heavy atoms and assessed the mDCC values of all-against-all pairs of these modes. We summarized them with a representative mDCC value for each pair of residues. We chose the largest mDCC value in the pair of residues, as follows:

$$mDCC\left(a,b\right)=\underset{i\in a,j\in b,k,l}{\text{max}}\left(mDCC\left(i,j;k,l\right)\right),$$ (6)

where the *i*-th and *j*-th atoms belong the *a*-th and *b*-th residues, respectively. In addition, we omitted the pairs that only rarely exist (*w*_*i*,*j;k*,*l*_(t) ≤ 0.1).

In order to investigate the propagations of the effects from the partner TFs to Ets1 via DNA, we particularly focused on the connections of positively correlated and directly contacting residue pairs, by using a network diagram. Residue pairs with an mDCC value equal or greater than 0.5 and a distance between the centers of modes less than 5 Å are connected with edges. Furthermore, the importance of each residue in the network was measured based on the “centrality” concept of the network. The betweenness, which is one of the centrality measurements, of the *i*-th residue is defined as:

$$g\left(i\right)=\sum_{s\neq i,t\neq i,s\neq t}^{}\frac{\sigma_{st}\left(i\right)}{\sigma_{st}}$$ (7)

where *σ*_*st*_ means the number of shortest paths between the *s*-th and *t*-th residues, and *σ*_*st*_*(i)* means the number of these paths that also include the *i*-th residue. Higher betweenness values imply that the residues are important to establish the interaction network.

### Mutagenesis assay

The cooperative effects of Runx1 on Ets1–DNA binding were experimentally assessed by electrophoretic mobility shift assays, for the wild-type and S332A-mutated Ets1. Details are described in S1 Text.

## Supporting information

S1 Text Experimental methods. Details of the mutagenesis experiments.
(PDF)

S2 Text Alternative parameter setting for network analysis. Details of the betweenness analysis with the alternative threshold of the mDCC value.
(PDF)

S1 Fig The time courses of the RMSD values. Each trajectory was superimposed on the backbones of all macromolecules in the system, and the RMSD values from the crystal structure were calculated for the backbone atoms. The red, blue, purple, green, and cyan plots represent (i) the quaternary complex, (ii) the Ets1–DNA complex, (iii) the isolated DNA, (iv) the Runx1 K176A model, and (v) the Ets1 E329A model, respectively. Panels (A), (B), (C), and (D) denote the RMSD values of Ets1, DNA, Runx1, and CBFβ, respectively.
(TIF)

S2 Fig The RMSF values for each residue of (A) Runx1 and (B) CBFβ. (TIF)

S3 Fig Gly333–C112 interaction in the quaternary complex. (A) The 3D structures around the Gly333–C112 interface at 305.0 ns (light green ribbons) and the crystal structure (grey ribbons). The red and blue meshes indicate the contours of the probability density distributions of the backbone nitrogen atom in Gly333 (Gly333:N) and the first oxygen atom in the phosphate group of C112 (C112:OP1), respectively. The probability density distributions were estimated by the mDCC analysis method (See Methods section). The red and blue spheres inside the meshes indicate the centers of the Gaussian functions, which are elements of the Gaussian mixture distributions. The distribution of the Gly333:N atom is modeled with the four Gaussian elements, with probabilities of 0.408, 0.384, 0.153, and 0.0496 marked as 1, 2, 3, and 4 in the figure, respectively. The distribution of C112:OP1 atom is modeled with three Gaussian elements, with probabilities of 0.756, 0.230, and 0.013, marked as 1, 2, and 3 in the figure. (B) The time course of the distance between Gly333:N and C112:OP1 in the trajectories of the quaternary complex (red) and the Ets1–DNA complex (blue).
(TIF)

S4 Fig The time courses of PC1 (A) and PC2 (B) for the quaternary complex (red) and the Ets1–DNA complex (blue). The vertical lines at 14.4 ns, 196.4 ns, and 305.0 ns correspond to the three representative structures of clusters 1, 2, and 3, respectively, in Fig 2.
(TIF)

S5 Fig Interactions between Ser332 and DNA. (A, B) Time courses of the interatomic distances of Ser332:C—C112:C5’ (A) and Ser332:Oγ–T113:OP1 (B). The red and blue plots are the results for the quaternary complex and the Ets1–DNA complex. (C) The 3D structures of the quaternary complex at 305.0 ns. The red and blue meshes indicate the contours of the probability density functions of the Ser332:C and C112:C5’ atoms, respectively. The spheres are the centers of the Gaussian functions for these probability density functions, with probabilities of 0.416, 0.338, 0.110, 0.0741, and 0.0621 for the elements marked 1, 2, 3, 4, and 5, respectively.
(TIF)

S6 Fig Quantitative EMSA. (A) Representative EMSA images for the binding of the wild-type (upper) and S332A-mutated (bottom) Ets1 fragments to the *TCRα* enhancer DNA, in the absence (left half in each gel image) or presence (right half in each gel image) of Runx1. The asterisk indicates shifted bands of minor contaminants from the purified S332A-mutated Ets1 fragment sample. (B) The quantified densities of the shifted bands of the Ets1-DNA complex fractions were plotted as mean ± standard deviation against the Ets1 concentrations, and fitted to a 1:1 binding model by the least squares method.
(TIF)

S7 Fig Structures of the RM. (A, B) Time courses of the interatomic distances of Ile321:Cγ–Ile429:Cγ (A) and Ile321:Cγ–Tyr424:Cζ (B) The red and blue plots show the results of the quaternary complex and the Ets1–DNA complex, respectively. (C, D) Snapshots of the RM, consisting of Val320–Gln336 and Val415–Val435: the crystal structure (C) and the snapshot at 305.0 ns (D).
(TIF)

S8 Fig Correlations between the HI2–H1 loop and the DNA. The plots indicate the maximum values of mDCC between each base and the residues in the HI2–H1 loop, Gly331 through Gln336. The red and blue lines are the results from the quaternary complex and the Ets1–DNA complex, respectively.
(TIF)

S9 Fig The interaction network of the Ets1–DNA complex model. See the legend of Fig 5.
(TIF)

S1 Table Details of simulation models. (PDF)

S2 Table Betweenness analysis. The top 15 high betweenness residues in each molecule in the quaternary complex. The betweenness values were calculated from the correlation network, created by the criteria mDCC ≥0.5 and distance between centers of Gaussian functions <5 Å (Fig 4).
(PDF)

S3 Table Betweenness analysis with the alternative network criteria. The top 15 high betweenness residues calculated with the different criteria from Supplementary S2 Table, *i*.*e*., mDCC ≥0.7 with the same distance threshold. The columns marked with ‘*’ indicate the ranks of the residues in S2 Table.
(PDF)

S1 Movie Conformational transitions of the HI2–H1 loop. The ribbons colored red, yellow, green, blue, and purple represent Ets1, CBFβ, Runx1, and the first and second strands of the DNA. Ser332 and C112 are depicted as sticks.
(MP4)

S2 Movie Conformational transitions of the RM, consisting of Val320–Gln336 and Val415–Val435. (MP4)

S3 Movie The global motion of the quaternary complex during the 400 ns trajectory. The ribbons are colored with the same scheme as in Fig 3A.
(MP4)
