# Supplementary material for: Molecular mechanisms of cooperative binding of transcription factors Runx1–CBFβ–Ets1 on the TCRα gene enhancer
Source: PLoS One. 2017 Feb 23;12(2):e0172654. doi: 10.1371/journal.pone.0172654 (PMC5322934; doi:10.1371/journal.pone.0172654)
Supplement: S1 Text — Details of the mutagenesis experiments. (PDF) [file pone.0172654.s001.pdf]

S1 Text. Experimental methods

Electrophoretic mobility shift assays (EMSA) were performed using the wild-type and S332A-mutated Ets1 fragments (amino acids 276–441, human) and the wild-type Runx1 fragment (a.a. 60–263, mouse), as previously described [1,2], to verify the MD simulation data suggesting the role of the interaction between the Ser332 side chain of Ets1 and the DNA phosphate backbone in the cooperative DNA binding of Ets1 with Runx1. Briefly, each of the three fragments of Ets1 (wild-type and S332A) and Runx1 (wild-type) described above was expressed in *E. coli* BL21(DE3) and purified by multiple column chromatography steps. The obtained samples were buffer-exchanged into a distilled water solution, containing 200 mM NaCl and 10 mM DTT for the Ets1 fragments, and that containing 10 mM DTT for the Runx1 fragment (high concentration of DTT is required to avoid oxidation of Runx1 fragment, which is quite sensitive to oxidation) [3]. The 30-bp DNA fragment containing the Runx1–Ets1 composite binding site from the *TCRα* enhancer [2] was synthesized, labeled using [γ-^{32}P]ATP and T4 polynucleotide kinase, and subjected to gel-filtration to remove the excess ATP. Two-fold serially diluted Ets1-containing solutions, ranging from 1.25×10^{-9} to 5.12×10^{-7} M, were mixed with the labeled DNA fragments in the absence of Runx1, while those ranging from 0.78×10^{-11} to 2.56×10^{-9} M were used in the presence of a constant concentration of Runx1 (2.0×10^{-8} M). The binding experiment was performed in reaction buffer [20 mM Tris-HCl (pH 7.2), 150 mM KCl, 1 mg/ml BSA, 10 mM DTT, 50 pg/μl poly(dI-dC), 0.005% Tween 20, and 2.5% Ficoll] at 25 °C for 20 min, and the resultant solutions were subjected to poly-acrylamide gel electrophoresis. The gels were analyzed on a Fuji BAS2500 image analyzer, and the band densities were quantified with the ImageGauge software (FujiFilm). The obtained data were plotted as the fraction of protein–DNA ([Ets1–DNA]/[total DNA] in the absence of Runx1 or [Ets1–Runx1–DNA]/[total DNA] in the presence of Runx1) against the Ets1 concentration [4-6]. The averaged values from three independent experiments were fitted to a 1:1 binding model by non-linear least square fitting, using the Gnuplot software package, to determine the dissociation constants. For the curve fitting values from the experiments using wild-type and S332A-mutated Ets1 in the absence of Runx1, six and nine data points in a lower concentration range were employed, respectively.

References

[1] M. Shiina, K. Hamada, T. Inoue-Bungo, M. Shimamura, S. Baba, K. Sato, et al., Crystallization of the Ets1–Runx1–CBFβ–DNA complex formed on the TCRα gene enhancer, Acta Crystallogr F Struct Biol Commun. 70 (2014) 1380–1384. doi:10.1107/S2053230X14018470/rl5078sup1.pdf.

[2] M. Shiina, K. Hamada, T. Inoue-Bungo, M. Shimamura, A. Uchiyama, S. Baba, et al., A Novel Allosteric Mechanism on Protein-DNA Interactions underlying the Phosphorylation-Dependent Regulation of Ets1 Target Gene Expressions, J. Mol. Biol. 427 (2015) 1655–1669. doi:10.1016/j.jmb.2014.07.020.

[3] T.H. Tahirov, T. Inoue-Bungo, M. Sasaki, A. Fujikawa, K. Kimura, K. Sato, et al., Crystallization and preliminary X-ray analysis of the C/EBPβ C-terminal region in complex with DNA, Acta Crystallogr D Biol Crystallogr. 57 (2001) 854–856. doi:10.1107/S0907444901003912.

[4] T.L. Goetz, T.L. Gu, N.A. Speck, B.J. Graves, Auto-inhibition of Ets-1 is counteracted by DNA binding cooperativity with core-binding factor α2, Mol. Cell. Biol. 20 (2000) 81–90. doi:10.1128/MCB.20.1.81-90.2000.
[5] Y.-Y. Tang, B.E. Crute, J.J. Kelley, X. Huang, J. Yan, J. Shi, et al., Biophysical characterization of interactions between the core binding factor α and β subunits and DNA, FEBS Lett. 470 (2000) 167–172. doi:10.1016/S0014-5793(00)01312-0.
[6] Y.Y. Tang, J. Shi, L. Zhang, A. Davis, J. Bravo, A.J. Warren, et al., Energetic and functional contribution of residues in the core binding factor β (CBFβ) subunit to heterodimerization with CBFα, J. Biol. Chem. 275 (2000) 39579–39588. doi:10.1074/jbc.M007350200.
