# Supplementary material for: Molecular mechanisms of cooperative binding of transcription factors Runx1–CBFβ–Ets1 on the TCRα gene enhancer
Source: PLoS One. 2017 Feb 23;12(2):e0172654. doi: 10.1371/journal.pone.0172654 (PMC5322934; doi:10.1371/journal.pone.0172654)
Supplement: S2 Text — Details of the betweenness analysis with the alternative threshold of the mDCC value. (PDF) [file pone.0172654.s002.pdf]

S2 Text. Alternative Parameter Setting for Network Analysis

As the betweenness values depend on arbitrarily adjusted parameters, such as the threshold of mDCC values (mDCC ≥0.5 was used), we also tested the betweenness analysis with the threshold mDCC ≥0.7 (S3 Table). The numbers of residues within the top 15 highest betweenness values under both conditions are ten and four for Ets1 and Runx1, respectively. Among these residues, eight and three of them are functionally important residues described above, and only two (Tyr395 and Ser332 for Ets1) and one (Arg83 for Runx1) were absent in the alternative parameter setting. This result suggests that functionally important residues tend to be effectively highlighted, regardless of the adjustable parameter value.
