# Supplementary figures and images for: Molecular mechanisms of cooperative binding of transcription factors Runx1–CBFβ–Ets1 on the TCRα gene enhancer
Source: PLoS One. 2017 Feb 23;12(2):e0172654. doi: 10.1371/journal.pone.0172654 (PMC5322934; doi:10.1371/journal.pone.0172654)

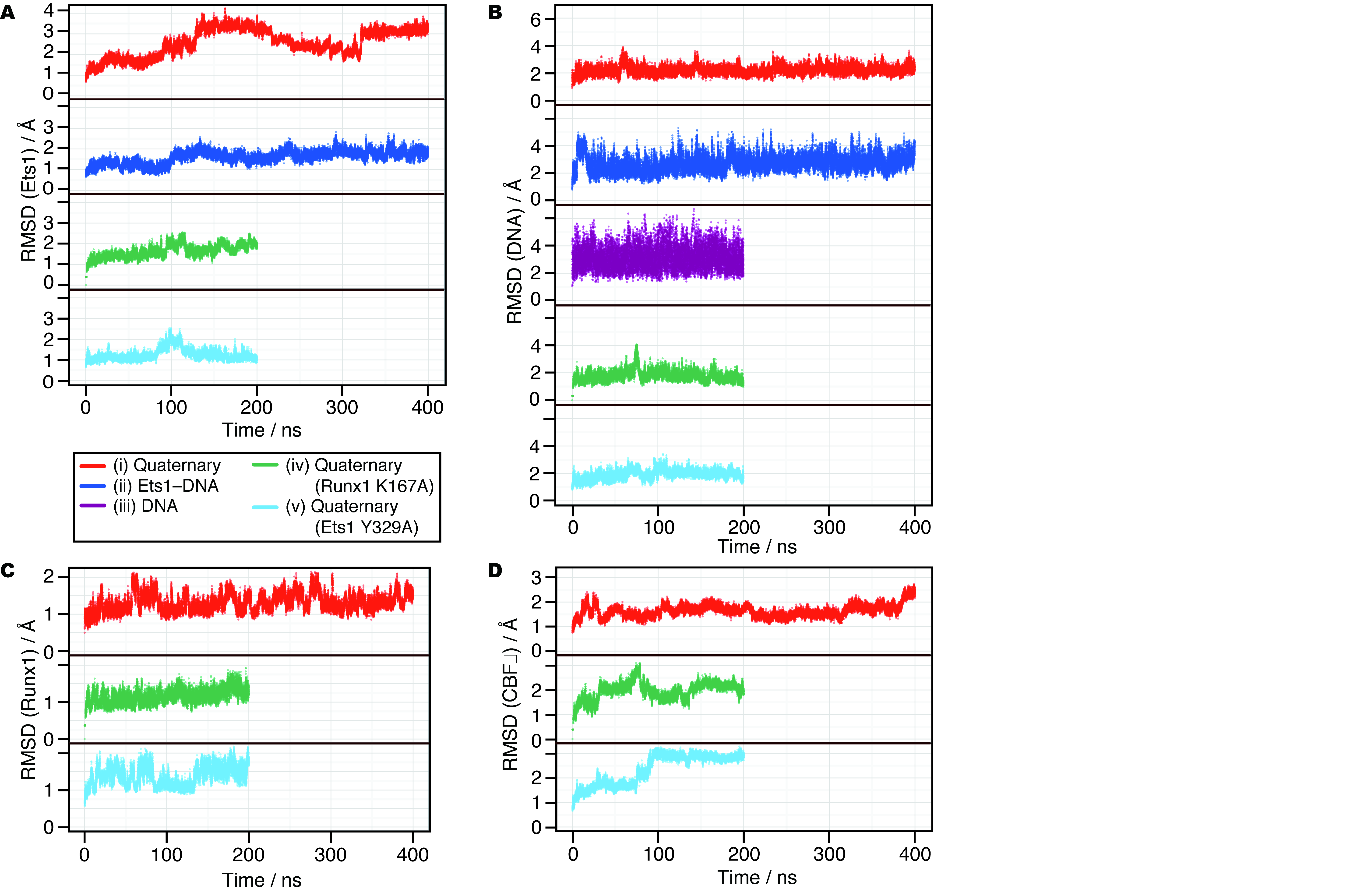

Supplement: S1 Fig — Each trajectory was superimposed on the backbones of all macromolecules in the system, and the RMSD values from the crystal structure were calculated for the backbone atoms. The red, blue, purple, green, and cyan plots represent (i) the quaternary complex, (ii) the Ets1–DNA complex, (iii) the isolated DNA, (iv) the Runx1 K176A model, and (v) the Ets1 E329A model, respectively. Panels (A), (B), (C), and (D) denote the RMSD values of Ets1, DNA, Runx1, and CBFβ, respectively. (TIF) [file pone.0172654.s003.tif]

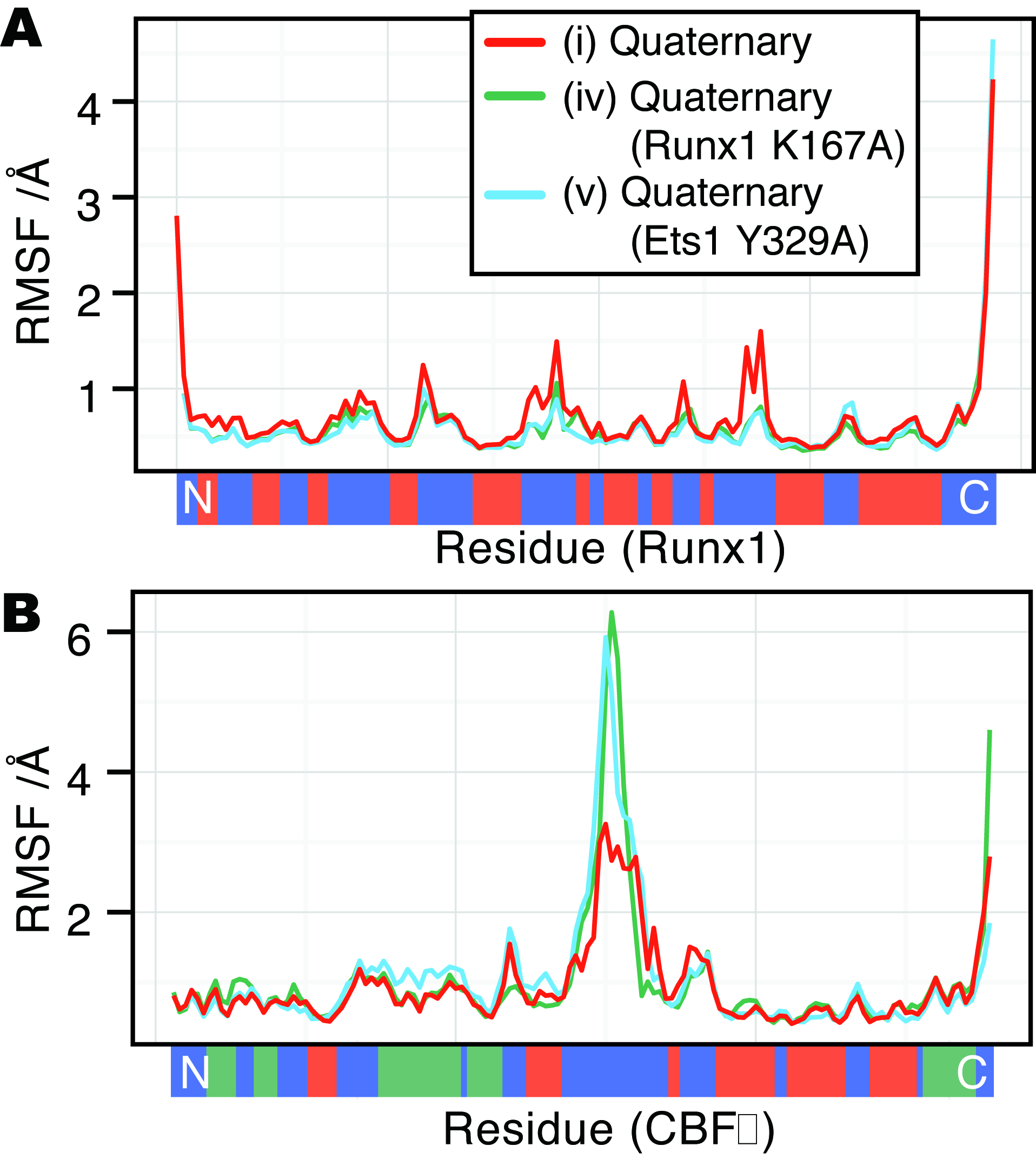

Supplement: S2 Fig — (TIF) [file pone.0172654.s004.tif]

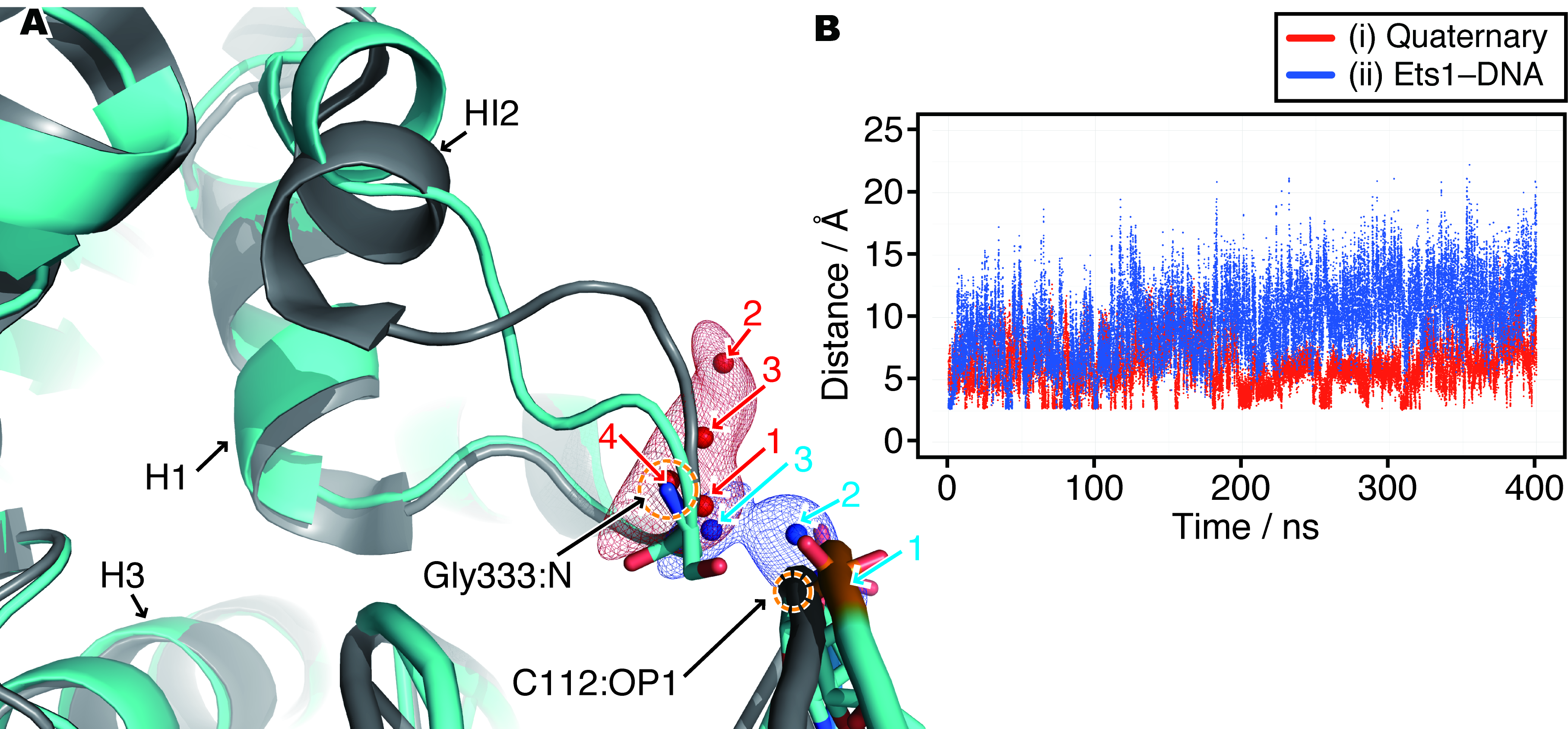

Supplement: S3 Fig — (A) The 3D structures around the Gly333–C112 interface at 305.0 ns (light green ribbons) and the crystal structure (grey ribbons). The red and blue meshes indicate the contours of the probability density distributions of the backbone nitrogen atom in Gly333 (Gly333:N) and the first oxygen atom in the phosphate group of C112 (C112:OP1), respectively. The probability density distributions were estimated by the mDCC analysis method (See Methods section). The red and blue spheres inside the meshes indicate the centers of the Gaussian functions, which are elements of the Gaussian mixture distributions. The distribution of the Gly333:N atom is modeled with the four Gaussian elements, with probabilities of 0.408, 0.384, 0.153, and 0.0496 marked as 1, 2, 3, and 4 in the figure, respectively. The distribution of C112:OP1 atom is modeled with three Gaussian elements, with probabilities of 0.756, 0.230, and 0.013, marked as 1, 2, and 3 in the figure. (B) The time course of the distance between Gly333:N and C112:OP1 in the trajectories of the quaternary complex (red) and the Ets1–DNA complex (blue). (TIF) [file pone.0172654.s005.tif]

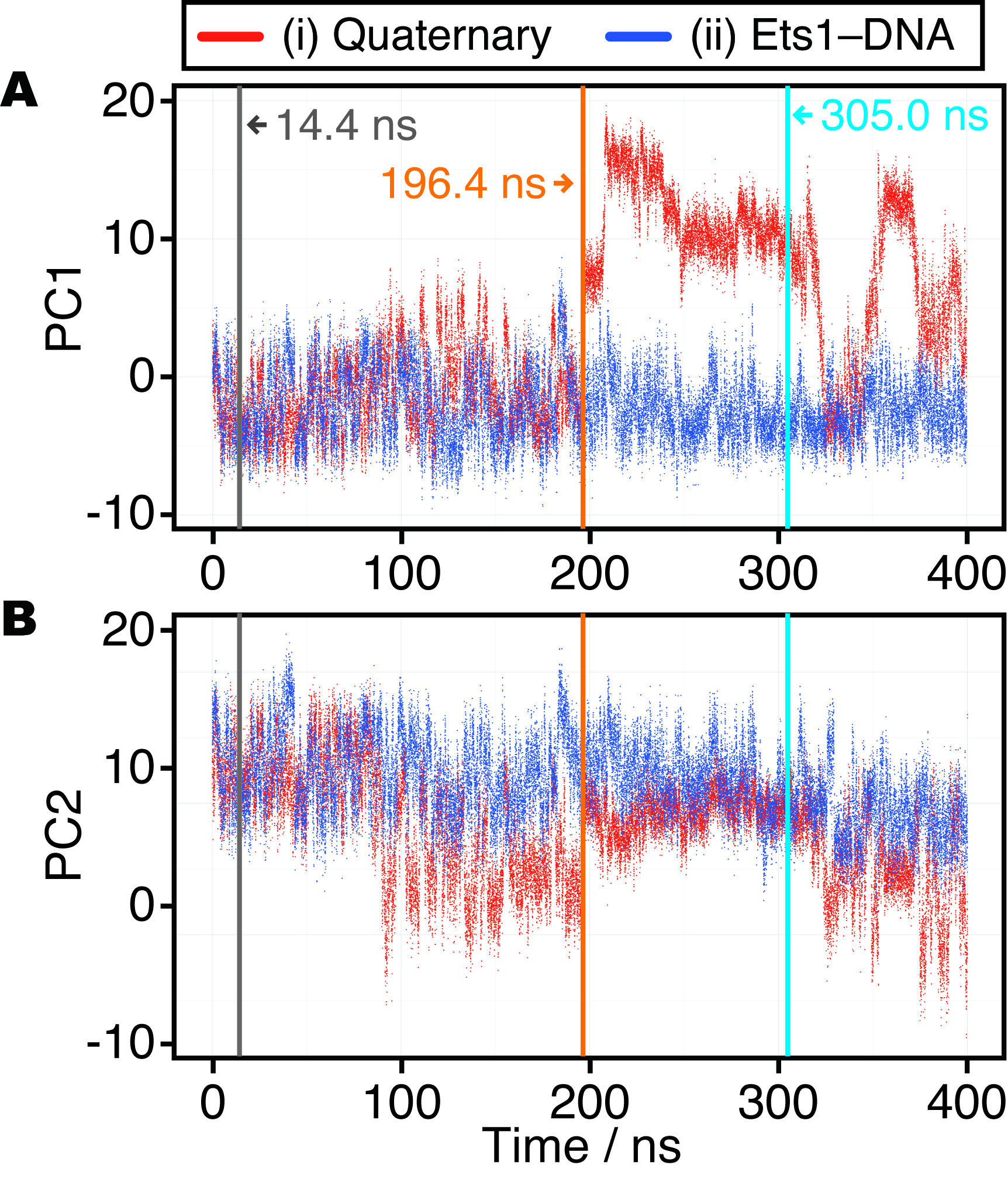

Supplement: S4 Fig — The vertical lines at 14.4 ns, 196.4 ns, and 305.0 ns correspond to the three representative structures of clusters 1, 2, and 3, respectively, in Fig 2. (TIF) [file pone.0172654.s006.tif]

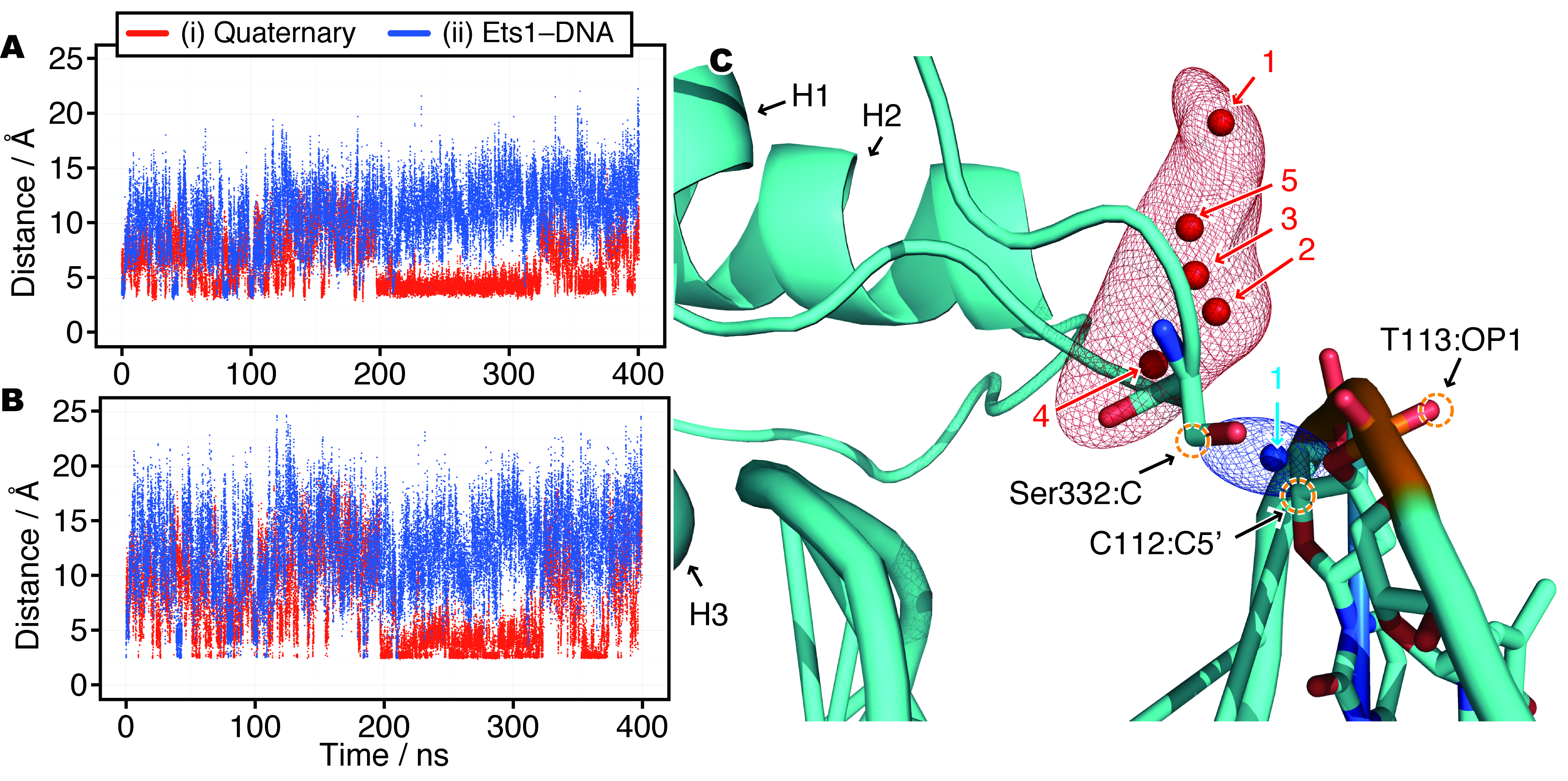

Supplement: S5 Fig — (A, B) Time courses of the interatomic distances of Ser332:C—C112:C5’ (A) and Ser332:Oγ–T113:OP1 (B). The red and blue plots are the results for the quaternary complex and the Ets1–DNA complex. (C) The 3D structures of the quaternary complex at 305.0 ns. The red and blue meshes indicate the contours of the probability density functions of the Ser332:C and C112:C5’ atoms, respectively. The spheres are the centers of the Gaussian functions for these probability density functions, with probabilities of 0.416, 0.338, 0.110, 0.0741, and 0.0621 for the elements marked 1, 2, 3, 4, and 5, respectively. (TIF) [file pone.0172654.s007.tif]

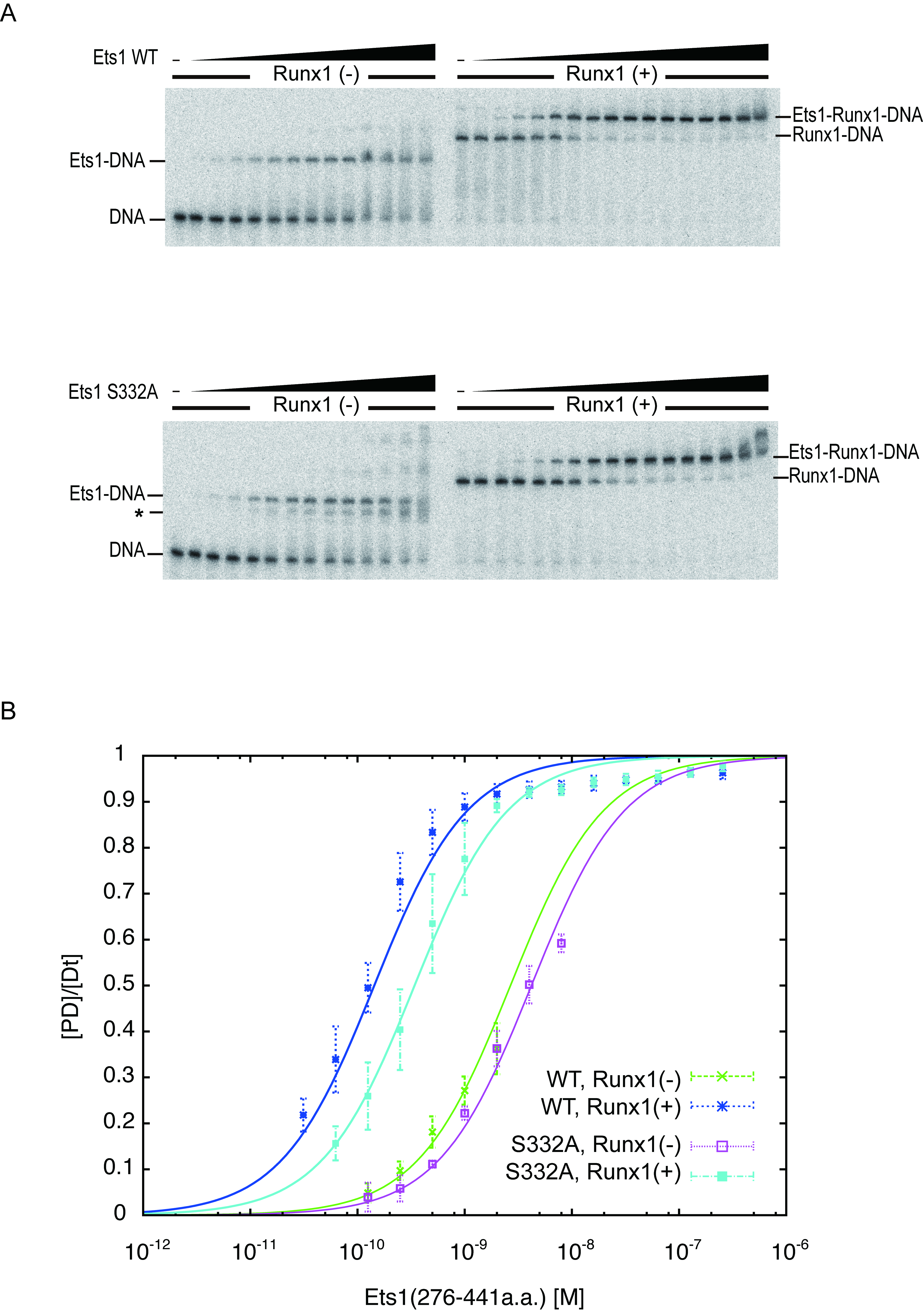

Supplement: S6 Fig — (A) Representative EMSA images for the binding of the wild-type (upper) and S332A-mutated (bottom) Ets1 fragments to the TCRα enhancer DNA, in the absence (left half in each gel image) or presence (right half in each gel image) of Runx1. The asterisk indicates shifted bands of minor contaminants from the purified S332A-mutated Ets1 fragment sample. (B) The quantified densities of the shifted bands of the Ets1-DNA complex fractions were plotted as mean ± standard deviation against the Ets1 concentrations, and fitted to a 1:1 binding model by the least squares method. (TIF) [file pone.0172654.s008.tif]

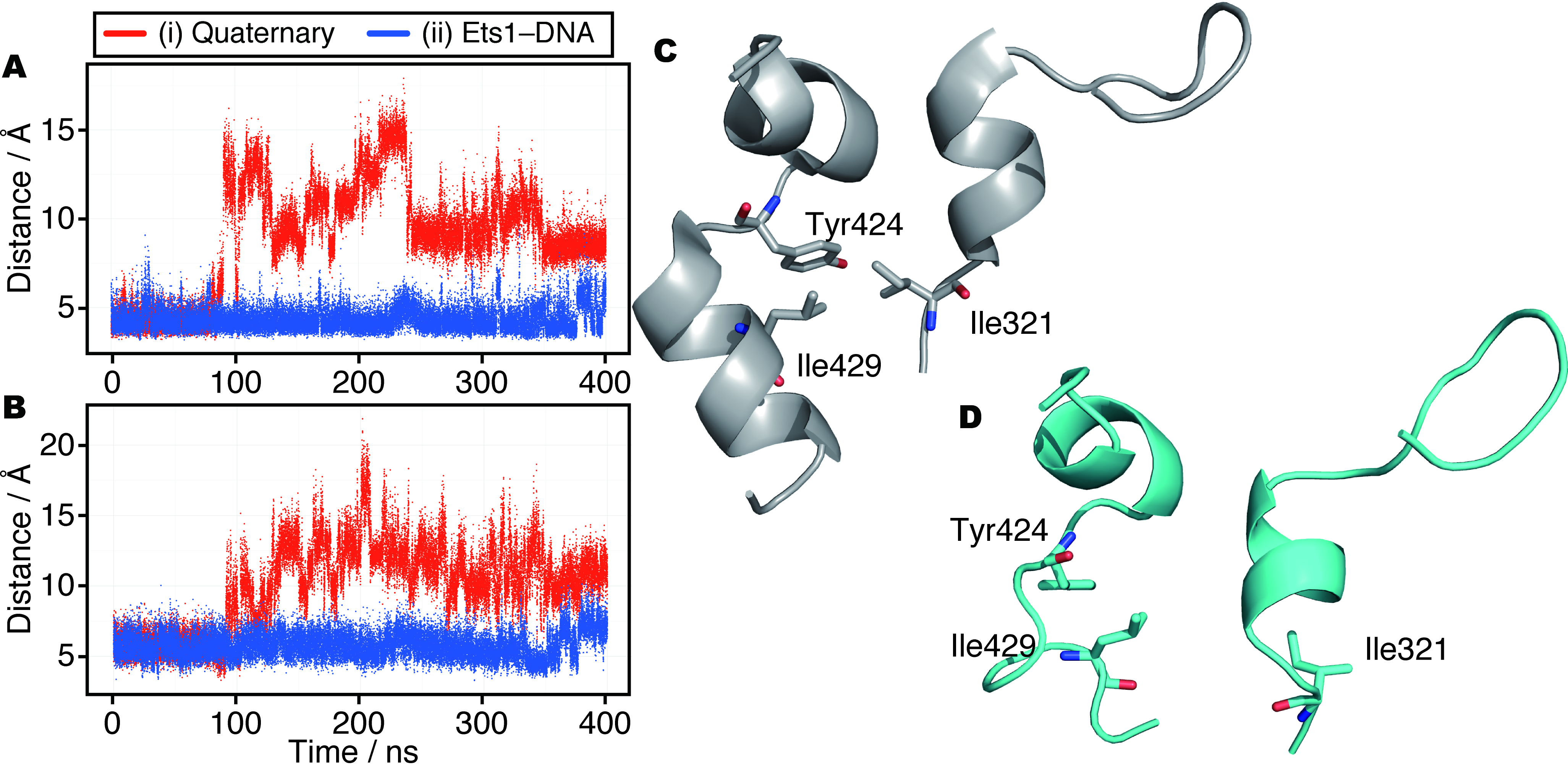

Supplement: S7 Fig — (A, B) Time courses of the interatomic distances of Ile321:Cγ–Ile429:Cγ (A) and Ile321:Cγ–Tyr424:Cζ (B) The red and blue plots show the results of the quaternary complex and the Ets1–DNA complex, respectively. (C, D) Snapshots of the RM, consisting of Val320–Gln336 and Val415–Val435: the crystal structure (C) and the snapshot at 305.0 ns (D). (TIF) [file pone.0172654.s009.tif]

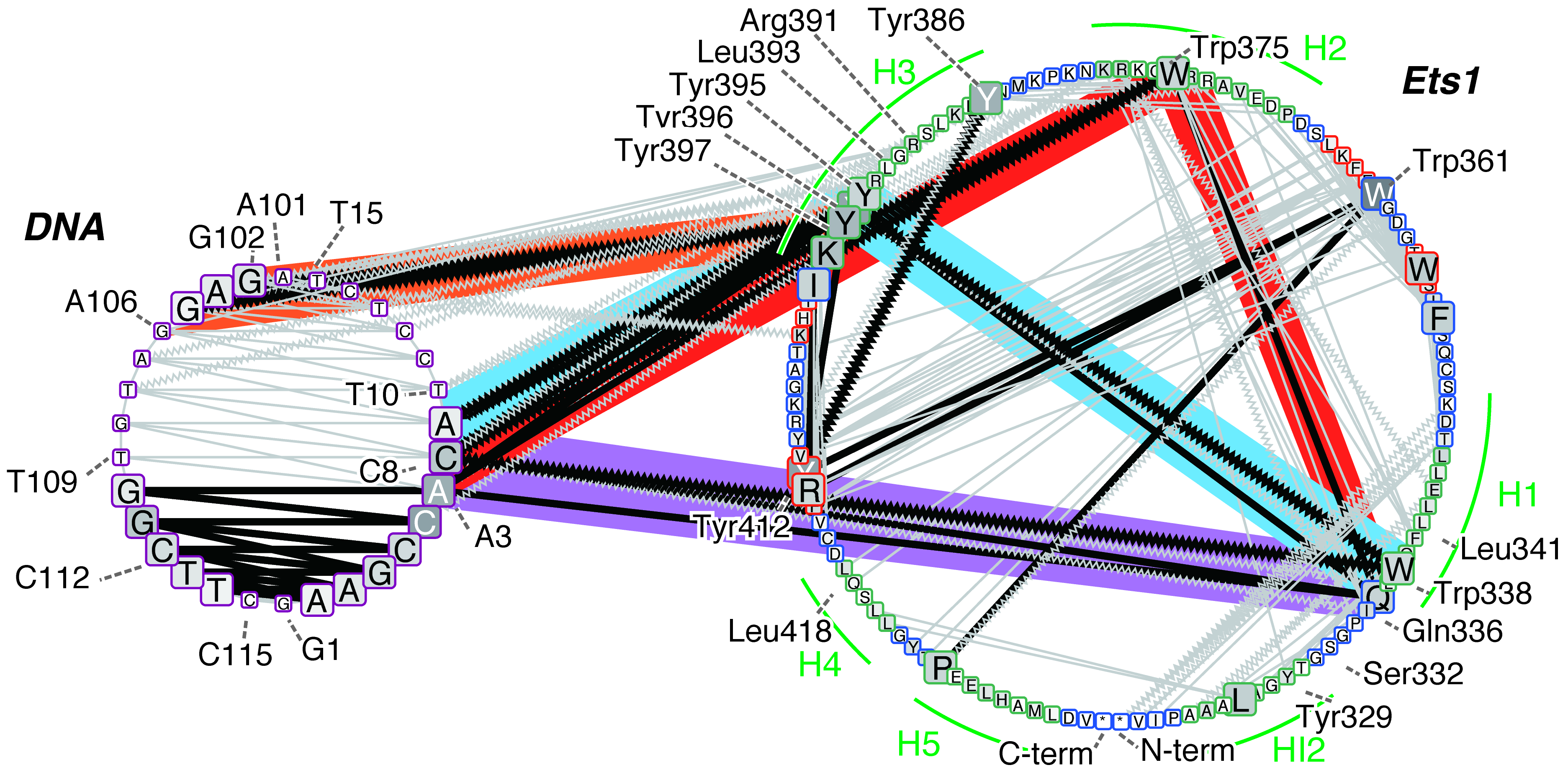

Supplement: S9 Fig — See the legend of Fig 5. (TIF) [file pone.0172654.s011.tif]
