# Supplementary material for: Molecular mechanisms of cooperative binding of transcription factors Runx1–CBFβ–Ets1 on the TCRα gene enhancer
Source: PLoS One. 2017 Feb 23;12(2):e0172654. doi: 10.1371/journal.pone.0172654 (PMC5322934; doi:10.1371/journal.pone.0172654)
Supplement: S1 Table — (PDF) [file pone.0172654.s012.pdf]

S1 Table. Details of simulation models.

	Model	# Atoms	# Waters	# Na^+ ions	# Cl^- ions	Simulation time /ns	Cell size /nm^3
i)	Runx1/CBFβ/Ets1/DNA	160,308	51,038	150	128	400	11.73^3
ii)	Ets1/DNA	63,788	20,319	67	43	400	8.623^3
iii)	DNA	60,407	19,783	68	40	200	8.480^3
iv)	K167A(Runx1)	163,722	52,180	154	131	200	11.81^3
v)	Y329A(Ets1)	159,548	50,851	149	127	200	11.70^3
