# Supplementary material for: Molecular mechanisms of cooperative binding of transcription factors Runx1–CBFβ–Ets1 on the TCRα gene enhancer
Source: PLoS One. 2017 Feb 23;12(2):e0172654. doi: 10.1371/journal.pone.0172654 (PMC5322934; doi:10.1371/journal.pone.0172654)
Supplement: S2 Table — The top 15 high betweenness residues in each molecule in the quaternary complex. The betweenness values were calculated from the correlation network, created by the criteria mDCC ≥0.5 and distance between centers of Gaussian functions <5 Å (Fig 4). (PDF) [file pone.0172654.s013.pdf]

S2 Table. Betweenness analysis.

	Runx1			CBFβ	
	Residue	Betweenness		Residue	Betweenness
1	Arg135	0.176	1	Leu64	0.0518
2	Leu134	0.116	2	Trp110	0.0501
3	Leu117	0.0994	3	Arg33	0.0476
4	Met106	0.0933	4	Arg40	0.0357
5	Arg80	0.0863	5	Phe57	0.0331
6	Phe146	0.0776	6	Asn104	0.0328
7	Lys83	0.0769	7	Ile55	0.0323
8	Glu116	0.0752	8	Ser65	0.0267
9	Val105	0.0636	9	Asn63	0.0258
10	Phe136	0.0591	10	Lys28	0.0251
11	Tyr162	0.0495	11	Arg3	0.0235
12	Arg139	0.0461	12	Thr60	0.0232
13	Ile150	0.0449	13	Arg131	0.0206
14	Phe153	0.0448	14	Phe44	0.0189
15	Val103	0.0441	15	Pro100	0.0184
	**Ets1**			**DNA**	
	Residue	Betweenness		Residue	Betweenness
1	Arg391	0.0830	1	A106	0.144
2	Trp338	0.0692	2	C8	0.0882
3	Tyr395	0.0487	3	A7	0.0852
4	Leu393	0.0432	4	G105	0.0734
5	Tyr396	0.0417	5	T107	0.0641
6	Ser332	0.0414	6	C112	0.0439
7	Tyr412	0.0356	7	G4	0.0398
8	Trp375	0.0339	8	A9	0.0389
9	Tyr329	0.0295	9	C6	0.0338
10	Gln336	0.0272	10	T10	0.0220
11	Tyr397	0.0257	11	A3	0.0198
12	Tyr386	0.0244	12	G104	0.0187
13	Leu341	0.0232	13	T109	0.0169
14	Trp361	0.0211	14	C5	0.0165
15	Leu418	0.0180	15	G102	0.0140

The top 15 high betweenness residues in each molecule in the quaternary complex. The betweenness values were calculated from the correlation network, created by the criteria mDCC ≥0.5 and distance between centers of Gaussian functions <5 Å (Fig 4).
