# Supplementary material for: Molecular mechanisms of cooperative binding of transcription factors Runx1–CBFβ–Ets1 on the TCRα gene enhancer
Source: PLoS One. 2017 Feb 23;12(2):e0172654. doi: 10.1371/journal.pone.0172654 (PMC5322934; doi:10.1371/journal.pone.0172654)
Supplement: S3 Table — The top 15 high betweenness residues calculated with the different criteria from Supplementary S2 Table, i.e., mDCC ≥0.7 with the same distance threshold. The columns marked with ‘*’ indicate the ranks of the residues in S2 Table. (PDF) [file pone.0172654.s014.pdf]

S3 Table. Betweenness analysis with the alternative network criteria

*	Runx1		*	CBFβ	
	Residue	Betweenness		Residue	Betweenness
51	Ala107	0.265	9	Asn63	0.250
6	Phe146	0.240	7	Ile55	0.0751
12	Arg139	0.167	21	Ile102	0.0555
1	Arg135	0.156	23	Thr30	0.0526
5	Arg80	0.101	24	Lys11	0.0479
29	Ala120	0.0897	27	Ile114	0.0454
69	Arg118	0.0889	2	Trp110	0.0425
20	Ile168	0.0594	46	Ala56	0.0414
37	Thr104	0.0568	8	Ser65	0.0392
39	Pro76	0.0521	38	Ile27	0.0379
50	Asp66	0.0513	66	Val4	0.0315
11	Tyr162	0.0502	15	Pro100	0.0311
48	Asp133	0.0500	20	Gln67	0.0287
43	Val74	0.0486	4	Arg40	0.0284
17	Phe131	0.0481	56	Ala99	0.0262
*	Ets1		*	DNA	
	Residue	Betweenness		Residue	Betweenness
5	Tyr396	0.0983	3	A7	0.186
1	Arg391	0.0685	9	C6	0.166
10	Gln336	0.0602	2	C8	0.0987
8	Trp375	0.0575	18	G108	0.0965
2	Trp338	0.0563	4	G105	0.0759
7	Tyr412	0.0493	1	A106	0.0747
12	Tyr386	0.0387	8	A9	0.0674
14	Trp361	0.0344	12	G104	0.0267
32	Ala327	0.0310	7	G4	0.0265
11	Tyr397	0.0306	14	C5	0.0175
21	Leu342	0.0278	23	G110	0.0130
24	Ile402	0.0270	11	A3	0.0120
28	Gln339	0.0247	15	G102	0.0103
36	Phe340	0.0242	16	A103	0.0099
4	Leu393	0.0215	5	T107	0.0097

The top 15 high betweenness residues calculated with the different criteria from S2 Table , *i.e.*, mDCC ≥0.7 with the same distance threshold. The columns marked with '*' indicate the ranks of the residues in S2 Table.
